# Trehalose dimycolate interferes with FcγR-mediated phagosome maturation through Mincle, SHP-1 and FcγRIIB signalling

**DOI:** 10.1371/journal.pone.0174973

**Published:** 2017-04-06

**Authors:** Emmanuel C. Patin, Anna C. Geffken, Sam Willcocks, Christoph Leschczyk, Albert Haas, Falk Nimmerjahn, Roland Lang, Theresa H. Ward, Ulrich E. Schaible

**Affiliations:** 1 Department of Immunology and Infection, Faculty of Infectious and Tropical Disease, London School of Hygiene and Tropical Medicine, London, United Kingdom; 2 Department of Molecular Infection Biology, Research Center Borstel, Borstel, Germany; 3 German Center for Infection Research, Borstel, Germany; 4 Institute for Cell Biology, University of Bonn, Bonn, Germany; 5 Division of Genetics, Department of Biology, University of Erlangen-Nuremberg, Erwin- Germany; 6 Institute of Clinical Microbiology, Immunology and Hygiene, University Hospital Erlangen, Friedrich-Alexander-Universität Erlangen-Nürnberg, Erlangen, Germany; Institut de Pharmacologie et de Biologie Structurale, FRANCE

## Abstract

The causative agent of tuberculosis, *Mycobacterium tuberculosis (M*. *tuberculosis)*, contains an abundant cell wall glycolipid and a crucial virulence factor, trehalose-6,6’-dimycolate (TDM). TDM causes delay of phagosome maturation and thus promotes survival of mycobacteria inside host macrophages by a not fully understood mechanism. TDM signals through the Monocyte-INducible C-type LEctin (Mincle), a recently identified pattern recognition receptor. Here we show that recruitment of Mincle by TDM coupled to immunoglobulin (Ig)G-opsonised beads during Fcγ receptor (FcγR)-mediated phagocytosis interferes with phagosome maturation. In addition, modulation of phagosome maturation by TDM requires SH2-domain-containing inositol polyphosphate 5’ phosphatase (SHP-1) and the FcγRIIB, which strongly suggests inhibitory downstream signalling of Mincle during phagosome formation. Overall, our study reveals important mechanisms contributing to the virulence of TDM.

## Introduction

The mycobacterial glycolipid trehalose-6,6’- dimycolate (TDM) is an abundant component of the cell wall of all *Mycobacterium* species [1]. In *Mycobacterium tuberculosis* (*M*. *tuberculosis*), the main causative agent of tuberculosis (TB) in humans, TDM is a potent virulence factor involved in establishing the intracellular niche crucial for mycobacterial survival and growth in macrophages [1]. Reconstitution of previously delipidated *M*. *tuberculosis* with purified TDM is sufficient to temporarily restore ability to delay phagosome acidification and fusion with lysosomes, thereby promoting bacterial survival [2]. Simplified bead models of phagosome biogenesis revealed the ability of purified mycobacterial and nocardial TDM to decelerate phagosome maturation and delay fusion with lysosomes [2–4]. The germline-encoded patter recognition receptor (PRR) monocyte-inducible C-type lectin (Mincle), a member of the C-type lectin receptor (CLR) family, plays an important role in immunity to mycobacterial and fungal pathogens [5,6]. Mincle has been reported as specific receptor for TDM [7,8]. Upon TDM binding, Mincle triggers the production of pro- and anti-inflammatory mediators such as tumour necrosis factor (TNF)-α, nitric oxide (NO) and IL-10 in murine bone marrow-derived macrophages (BMDM) through the Syk/CARD9 signalling axis [9–12]. However, little is known about its role in phagosome biogenesis. Fcγ receptors (FcγR) are important phagocytic receptors, which mediate phagocytosis of immunoglobulin (Ig)G-opsonised particles. Signal transduction depends on cytosolic domain containing immunoreceptor tyrosine-based activation (ITAM) or immunoreceptor tyrosine-based inhibition (ITIM) motifs for activating or inhibitory FcγR, respectively [13]. Both Mincle and activating FcγR associate with the transmembrane adaptor protein Fcγ chain containing an ITAM and initiating signalling by Syk recruitment [10]. Mycobacteria are potent immunogens inducing specific antibodies to surface molecules, which can be involved in phagocytic uptake of *M*. *tuberculosis* [14]. Phagocytes usually rapidly eliminate IgG-opsonised bacteria by FcγR-mediated phagocytosis, which is involved in host defence to *M*. *tuberculosis* as mice deficient in activating FcγRs were more susceptible to *M*. *tuberculosis* infection than WT ones. In contrast, mice lacking the inhibitory FcγRIIB were more resistant to experimental TB [15].

By employing IgG-opsonised beads as model to study FcγR-mediated phagosome biogenesis, we show herein that co-engaging Mincle and FcγR by its ligands, TDM and IgG, respectively, trigger an inhibitory cross talk, which decelerates maturation of phagosomes containing IgG-opsonised beads. TDM also requires both, SH2-domain-containing protein tyrosine phosphatase (SHP-1) and the inhibitory FcγRIIB to delay phagosome maturation. Thus, our study reveals that TDM uses an innate immune receptor along with inhibitory signalling to down-regulate phagosome biogenesis.

## Results and discussion

### TDM impairs maturation of IgG-opsonised bead-phagosomes

**Fig 1:**
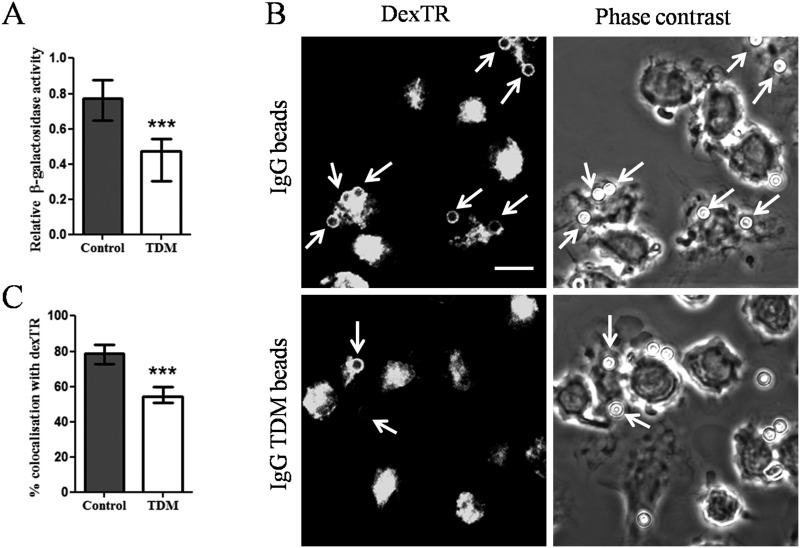
TDM inhibits maturation of IgG-coated bead-phagosomes. Raw 264.7 macrophages or dexTR-labelled BMDMs were incubated with IgG or IgG TDM beads (ratio 1:5) for 30 min. The graph (A) indicates the relative β-galactosidase activity from purified bead-phagosomes. The panel (B) gives representative confocal pictures for each bead sample with arrow-heads indicating dexTR-positive bead-phagosomes. Scale bar: 10 μm. The graph (C) indicates the quantification by CLSM of the percentage of bead-phagosomes colocalising with dexTR. Data (A and C) are expressed as medians of either relative β-galactosidase activity or % of dexTR-positive bead-phagosomes and combined from three (A) or six (C) independent experiments done in triplicate ± interquartile ranges (Mann-Whitney T-test: ***p<0.001).

Simplified bead models to study phagosome biogenesis revealed the ability of TDM from both, mycobacterial and closely related nocardial species, to decelerate bead phagosome maturation [2–4]. We therefore investigated whether TDM can affect FcγR-mediated phagosome biogenesis. IgG-opsonised beads with or without TDM (IgG vs. IgG TDM beads) were added to Raw 264.7 macrophages and 30 min later, bead-phagosomes were isolated [3]. IgG TDM-bead phagosomes (Fig 1A) contained significantly less activity of the lysosomal marker enzyme, β-galactosidase than control IgG bead-phagosome preparations. Uptake of IgG-opsonised beads was primarily FcγR-mediated as shown by a decrease in internalisation when cells were treated with an anti-FcγR antibody compared to untreated or isotype control treated ones (S1 Fig). Maturation of bead-phagosomes was additionally analysed for fusion with Dextran Texas Red (dexTR) labelled-lysosomes in BMDM using confocal laser scanning microscopy (CLSM). Lysosomal tracer acquisition by IgG TDM bead-phagosomes was significantly reduced 30 minutes after internalisation when compared with IgG control ones (Fig 1B and 1C).

Together, these results show that TDM is able to delay maturation of phagosomes containing IgG-opsonised beads, which mediate phagocytosis through FcγR. These results extend the previously observed ability of TDM to interfere with phagosome maturation in macrophages to FcγR-mediated phagosome biogenesis [2,3].

### TDM engages Mincle to interfere with maturation of IgG-opsonised bead-phagosomes

**Fig 2:**
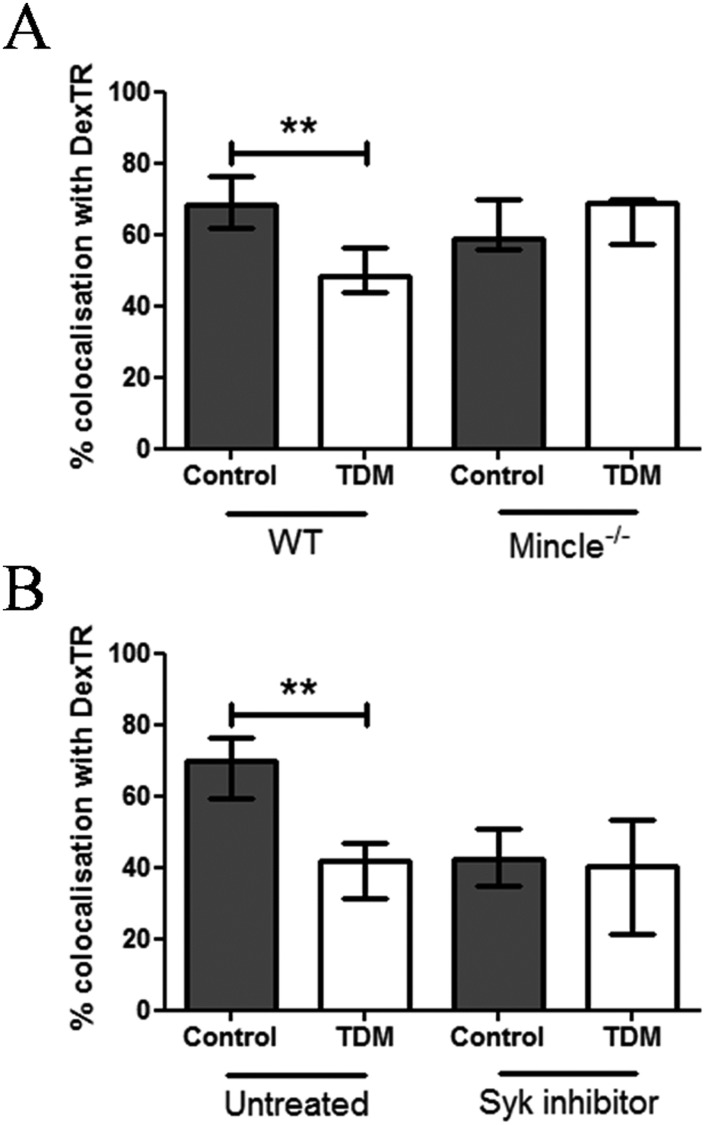
Mincle signalling inhibits IgG bead-phagosome maturation. (A) DexTR-labelled WT and Mincle KO BMDMs were incubated with IgG or IgG TDM (ratio 1:5) for 30 min. In (B), the same procedure as (A) was applied but WT BMDMs were treated with 200nM Syk inhibitor or medium for 30 min, prior to bead addition. The graphs show the quantification by CLSM of the percentage of bead-phagosomes colocalising with dexTR. Data are expressed as medians % of dexTR-positive bead-phagosomes and combined from three independent experiments done in triplicate ± interquartile ranges (Kruskal-Wallis test followed by Dunns post-test: **p<0.01).

To address the question whether Mincle is involved in TDM-mediated deceleration of phagosome maturation upon FcγR-mediated phagocytosis, WT or Mincle^-/-^ BMDM whose lysosomes had been labelled with dexTR were incubated with IgG and IgG TDM beads. TDM-mediated inhibition of IgG bead-phagosome maturation was completely abolished in the absence of Mincle 30 min following internalisation (Fig 2A). IgG bead-phagosome maturation was temporarily delayed, rather than completely inhibited, by TDM, since 180 min after uptake, we observed similar percentage of IgG TDM beads colocalising with dexTR or containing enhanced β-galactosidase activity as compared with control ones (S2A and S2B Fig). Notably, adding TDM exogenously to macrophage cultures independent of beads (by coating the lipid to the coverslips) did not interfere with control bead phagosome biogenesis (S3 Fig). Therefore, inhibition of phagosome maturation by TDM requires the presence of the glycolipid at the bead-phagocyte interface to allow direct interaction with host cell targets such as Mincle. We can also not fully exclude that destabilisation of the proximal phagosomal membrane by bead-surface coated TDM can interfere with phagosome maturation.

As Mincle's pro-inflammatory function requires Syk signalling, trafficking of IgG TDM beads was analysed in the presence or absence of the Syk inhibitor imidazopyrimidine. We found that Syk inhibition interfered with maturation of phagosomes with IgG-opsonised beads as previously reported [16]. However, interference of TDM with IgG-opsonised bead phagosome biogenesis 30 min after uptake was not altered by Syk inhibition (Fig 2B). Thus, Syk drives FcγR-mediated phagosome maturation, whereas TDM interaction with Mincle interferes with FcγR/Syk-mediated phagosome maturation. We can also suggest that inhibition of Syk simply blocks phagocytosis not permitting further down-regulation of IgG-mediated phagosome maturation by TDM. Despite Mincle is not categorised as phagocytic receptor our findings strongly suggest that Mincle has a regulatory function when co-engaged during FcγR-mediated phagocytosis. In addition, a recent study reported a defect in Mincle-deficient neutrophils to phagocytose Gram-negative bacteria, such as *Klebsiella pneumoniae* [17]. Mincle is only expressed at low levels in resting macrophages whereas enhanced expression requires activating signals such as those by TLR ligands or interferon gamma [9,18]. We recently showed that *M*. *bovis* BCG can promote Mincle expression *in vitro* [12]. Nevertheless, we cannot rule out that the macrophage C-type lectin, MCL, recently described as additional but constitutively expressed TDM receptor required to prime Mincle activity, also participates in phagosome biogenesis [19]. In the absence of antibodies, TDM failed to reduce BSA-bead phagosome maturation when endotoxin-free BSA was used, an experimental set up different from Axelrod et al. (S4 Fig). Therefore, our data indicate that deceleration of phagosome maturation via TDM-Mincle interaction is only relevant upon FcγR recruitment.

Taken together, our results reveal a novel facet of TDM's virulence property, i.e. exploiting Mincle, an otherwise pro-inflammatory receptor, to delay phagosome maturation upon FcγR-mediated uptake, likely through co-recruitment of Mincle and FcγR associated regulatory pathways. Our findings suggest that TDM/Mincle interaction contributes to the poor protective efficacy of specific antibodies during *M*. *tuberculosis* infection.

### SHP-1 and FcγRIIB are required for TDM-induced impairment of FcγR-mediated phagosome biogenesis

**Fig 3:**
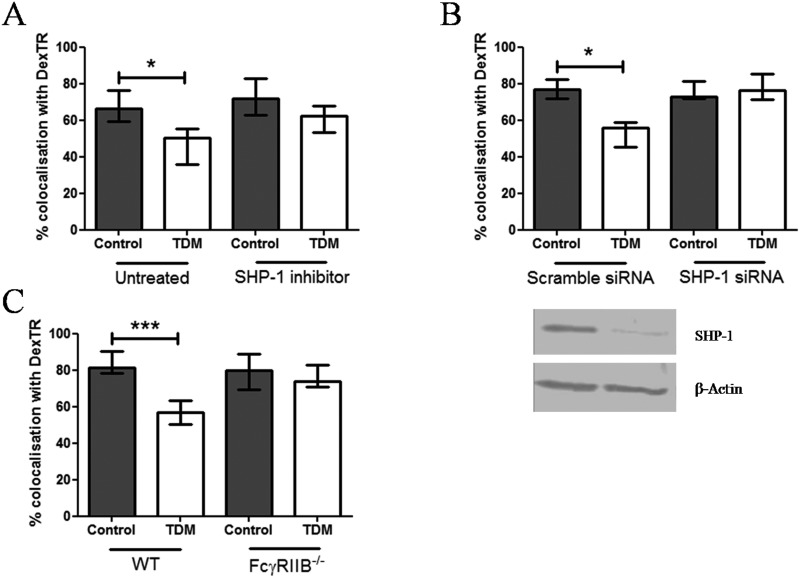
SHP-1 and FcγRIIB mediate impairment of IgG bead-phagosomes maturation by TDM. DexTR-labelled WT untreated (A, B and C), SHP-1 siRNA, scramble siRNA-transfected (B) or FcγRIIB KO (C) BMDMs were either treated with 11 μM SHP-1 inhibitor (A) prior incubation with IgG or IgG TDM beads (ratio 1:5) or were directly incubated with beads (B and C) for 30 min. (A, B and C) The graphs indicate quantification by CLSM of the percentage of bead-phagosomes colocalising with dexTR. (B) 2 μg of protein extracts from SHP-1 siRNA or scramble siRNA-transfected BMDMs were assessed for SHP-1 expression by Western blot (loading control: β-actin). Data are expressed as means % of dexTR-positive bead-phagosomes and combined from two (A and B) and three (C) independent experiments done in triplicate ± interquartile ranges (Kruskal-Wallis test followed by Dunns post-test: *p<0.05; *** *p*<0.001). Western blot (C) is representative for two independent experiments.

To identify inhibitory signalling molecules involved in the interference with FcγR-mediated phagosome maturation upon TDM-Mincle interaction, we analysed ITAM-containing adaptor molecules required for both, Mincle and FcγR signalling [10,20]. Therefore, we explored whether the ITAM phosphatase SHP-1 is a potential target of TDM-Mincle mediated interference with phagosome maturation. BMDM pre-labelled with dexTR and pre-treated with the SHP-1 inhibitor sodium stibogluconate [21] were challenged for 30 min with IgG or IgG TDM beads. Inhibition of SHP-1 abolished deceleration of IgG bead-phagosome maturation by TDM to a similar level as control IgG bead-phagosomes (Fig 3A). However, we observed no effect of SHP-1 inhibition on phagosome maturation of IgG-control beads, which demonstrate that TDM is crucial for triggering inhibitory signals in our model. Comparable to pharmacological inhibition, knock down of SHP-1 expression also abrogated TDM-mediated inhibition of FcγR-mediated phagosome maturation (Fig 3B). Our observations that SHP-1 controls phagosome biogenesis extend previous studies demonstrating that SHP-1 can inhibit FcγR-mediated phagocytosis [22–24]. Notably, SHP-1 is involved in IL-12p40 production by macrophages through inhibition of phosphatidylinositol-3 kinase (PI3K) [25]. PI3K leads to generation of phosphatidylinositol-3 phosphate (PI(3)P), a membrane lipid that recruits the early endosomal antigen-1 (EEA-1) involved in late phagosome formation [26]. We therefore hypothesise that TDM-Mincle interaction activates SHP-1 to subsequently inhibit PI3K and phagosome maturation. Our study corroborates and extends the observation by Iborra and colleagues, which recently revealed the link between Mincle and SHP-1 during *Leishmania* infection [27]. These authors demonstrated that recognition of the parasites by Mincle triggers an inhibitory FcγR-SHP-1 axis in dendritic cells subsequently leading to down-regulation of Th_1_ responses required for protective immunity to *Leishmania* parasites. Finally, our findings differ from a recent study showing that SHP-1 promotes non-opsonised bead-phagosome maturation [28] indicating that SHP-1 has differential regulatory functions depending on whether phagocytosis is mediated by FcγR or other receptors.

SHP-1 is recruited to the ITIM-bearing inhibitory FcγRIIB involved in the inhibition of FcγR-mediated phagocytosis [29]. We therefore investigated whether FcγRIIB is involved in TDM-mediated inhibition of IgG bead-phagosome maturation. BMDM from WT and FcγRIIB KO mice were challenged with IgG or IgG TDM beads and assessed for fusion with dexTR-labelled lysosomes 30 min following internalisation. Whereas fusion of IgG bead-phagosomes with lysosomes was inhibited by TDM in WT macrophages, the glycolipid failed to interfere with phagosome maturation in the absence of FcγRIIB (Fig 3C). The fact that maturation of control IgG bead-phagosomes is unaffected by absence of FcγRIIB excludes a direct interference with FcγR-mediated phagosome biogenesis through recruitment of the inhibitory receptor by IgG alone. These findings suggest a link of both, Mincle and FcγRIIB to the regulation of phagosome biogenesis upon FcγR-mediated uptake of IgG-opsonised particles. In support of this notion, IgG1 immune complexes have been shown to promote association between FcγRIIB and Dectin-1, another CLR, which share similarities with Mincle, thereby inhibiting C5a-mediated inflammatory properties [30]. The potential link between Mincle and FcγRIIB could explain the recent discovery that the inhibitory function of FcγRIIB is important for *M*. *tuberculosis* escape from host immunity [31].

Herein, we show that by IgG-opsonised TDM-coated particles recruit Mincle and FcγRIIB to induce signals that delay FcγR-mediated phagosome maturation. Moreover, SHP-1 is essential for the inhibitory pathway triggered by TDM suggesting a signalling cascade downstream of Mincle and SHP-1 alternative to the activating Syk/CARD9 one. Thus, our study revealed a novel facet of TDM virulence through involvement of Mincle and inhibitory downstream signalling.

## Materials and methods

### Ethical statement

All animal experiments were performed in accordance with the German Animal Protection Law and were approved by the Ethics Committee for Animal Experiments of the Ministry for Agriculture, Environment, and Rural Areas of the State of Schleswig-Holstein, Germany (Kommission für Tierversuche/Ethik-Kommission des Landes Schleswig-Holstein) under the license V312-72241.123–3 (24-2/07) or 102-8/09.

### Macrophages

BMDMs were differentiated from bone marrow cells of WT, Mincle or FcγRIIB KO C57BL/6 mice and the Raw 264.7 macrophage cell line was maintained as previously described [32].

### IgG-opsonised lipid-coated beads

Magnetic Tosyl-activated beads (Dynabeads M-280, Invitrogen, UK) covalently coated with sterile endotoxin-free bovine serum albumin (BSA, Sigma-Aldrich, UK) were coated with TDM from *M*. *tuberculosis* (Sigma-Aldrich, UK or a kind gift from Prof. Otto Holst from the Research Center Borstel, Germany) as previously described [3]. 5×10^7^ glycolipid-coated magnetic beads were incubated with 100 μg/ml mouse monoclonal anti-BSA antibody (Sigma-Aldrich, UK) for 1 h at RT or overnight at 4°C. Presence of TDM was confirmed by TLC as previously described (Axelrod et al., 2008).

### Assessment of phagosome maturation by the β-Galactosidase assay or CLSM

For β-Galactosidase assay, Raw 264.7 macrophages were seeded at 10^7^ cells/T75 flasks. Macrophages were incubated in D10 with opsonised beads at a multiplicity of 5 beads/cell. for 30 min at 37°C. Bead-phagosomes were isolated using magnetic separation as previously described [3,33]. Levels of β-Galactosidase activity were measured as previously described [34].

For CLSM, BMDMs were seeded at 10^5^ cells per well in triplicates on microscope chamber slides (Lab-Tek chambered coverglass II, Fisher Scientific, UK) in D10 supplemented with 100 μg/ml Dextran Texas Red (dexTR) (10 kDa; Invitrogen, UK) for 3 hours at 37°C and BMDMs were chased with fresh media, overnight at 37°C. Where indicated, macrophages were also incubated with 200 nM Syk inhibitor (imidazopyrimidine, Santa Cruz Biotechnology, USA) or 11 μM SHP-1 inhibitor (stibogluconate, Calbiochem, Germany) for 30 min at 37°C in D10 prior to bead addition. Macrophages were incubated in D10 in technical triplicates with opsonised beads at a multiplicity of 5 beads/cell. Phagocytosis was allowed to proceed for the indicated time in D10 at 37°C. Cells were fixed with 2% PFA for 15 min at RT. Chamber slides were mounted with an aqueous-based mounting medium (Fluoromount, Sigma-Aldrich, UK) for CLSM analysis. Fluorescence images were obtained with a Zeiss LSM 510 confocal microscope using the 543nm laser line with a 63X 1.3 NA objective set at pinhole diameter equivalent to 1–1.5 Airey units. Images were analysed using the Zeiss LSM software. Images were taken at 20x magnification. Following removal of the fluorescent background, percentages of beads co-localizing with lysosomes were determined by manually counting beads positive for dexTR staining in at least 50 macrophages per replicate.

### siRNA transfection of BMDMs

SHP-1 expression was knocked-down with specific siRNA using the Amaxa Mouse Macrophage Nucleofactor Kit (Lonza Group). 10^7^ BMDMs were transfected with 20 μg/ml SHP-1 siRNA (SMARTpool: siGENOME PTPN6 siRNA, Thermo Scientific Dharmacon; 5'-UGACAGAGCUGGUCGAGUA-3'; 5'-GAACAAAUGUGUCCCAUAC-3';5'-GCAAGAACCGCUACAAGAA-3'; 5'-UGACACAGCAGAAUACAAA-3') or control scramble siRNA (siGENOME non-targeting siRNAs, Thermo Scientific Dharmacon) following manufacturer's instructions. The assessment of SHP-1 expression knock-down was performed by Western blot using specific SHP-1 antibody (Santa Cruz, UK).

### Statistical analysis

Data are presented as means ± interquartile ranges and are combined from 2–6 independent experiments done in triplicate. Kruskal-Wallis test followed by Dunns post-test was used for statistical analysis when multiple groups were analyzed. Mann-Whitney test were used for statistical analysis when two groups were analyzed.

## Supporting information

S1 Fig Phagocytosis of IgG-opsonised beads is FcγR-mediated. Raw 264.7 macrophages were treated with or without either anti-FcγR antibody (2.4G2) or control isotype for 10 min followed by the addition of IgG beads (ratio 3:1) for 5 min. Non-internalised beads were differentiated from internalised beads using an anti-mouse Cy3 secondary antibody under non-permeabilising conditions. The graph shows quantifications by CLSM of the percentage of internal beads. Scale bar: 10 μm. Data are expressed as medians % of internalised beads and combined from two independent experiments ± interquartile ranges.
(TIF)

S2 Fig Maturation of IgG bead-phagosome is delayed by TDM. DexTR-labelled BMDMs (A) or Raw 264.7 (B) macrophages were incubated with IgG or IgG TDM beads (ratio 1:5) and phagocytosis was allowed to proceed for 10, 30 or 180 min. The graph (A) indicates the quantification by CLSM of the percentage of bead-phagosomes colocalising with dexTR. Beads were assessed in BMDMs for fusion with dexTR-labelled lysosomes by CLSM. The graph (B) indicates the relative β-galactosidase activity from purified bead-phagosomes. Data are expressed as medians % of dexTR-positive bead-phagosomes (A) or relative β-galactosidase activity (B) from a single experiment each done in triplicates ± interquartile ranges.
(TIF)

S3 Fig TDM exogenously added to the cultures fails to decelerate bead phagosome maturation. Coverslips were either coated with TDM (5 μg, equalizing 5x105 TDM coated beads) or left untreated before BMDM were seeded and labelled with DexTR. Macrophages were incubated with control BSA beads (ratio 1:5) for 30 min. The graph shows quantification by CLSM of the percentage of bead-phagosomes colocalising with dexTR. Data are expressed as medians % of dexTR-positive bead-phagosomes done in triplicates ± interquartile ranges.
(TIFF)

S4 Fig TDM does not affect the phagosome maturation of non-opsonised endotoxin free BSA beads. DexTR-labelled BMDMs macrophages were incubated with control BSA or TDM BSA beads (ratio 1:5) and phagocytosis was allowed to proceed for 30 min. The BSA used for coating beads in all experiments is endotoxin free. The graph indicates the quantification by CLSM of the percentage of bead-phagosomes colocalising with dexTR. Beads were assessed in BMDMs for fusion with dexTR-labelled lysosomes by CLSM. Data are expressed as medians % of dexTR-positive bead-phagosomes done in triplicates ± interquartile ranges.
(TIF)

S1 File Supplementary materials. Experimental procedures: 1. Assessment of bead phagocytosis; 2. Isolation of bead-phagosomes; 3. SHP-1 expression analysis by Western blot
(DOC)
