# Supplementary figures and images for: Trehalose dimycolate interferes with FcγR-mediated phagosome maturation through Mincle, SHP-1 and FcγRIIB signalling
Source: PLoS One. 2017 Apr 6;12(4):e0174973. doi: 10.1371/journal.pone.0174973 (PMC5383150; doi:10.1371/journal.pone.0174973)

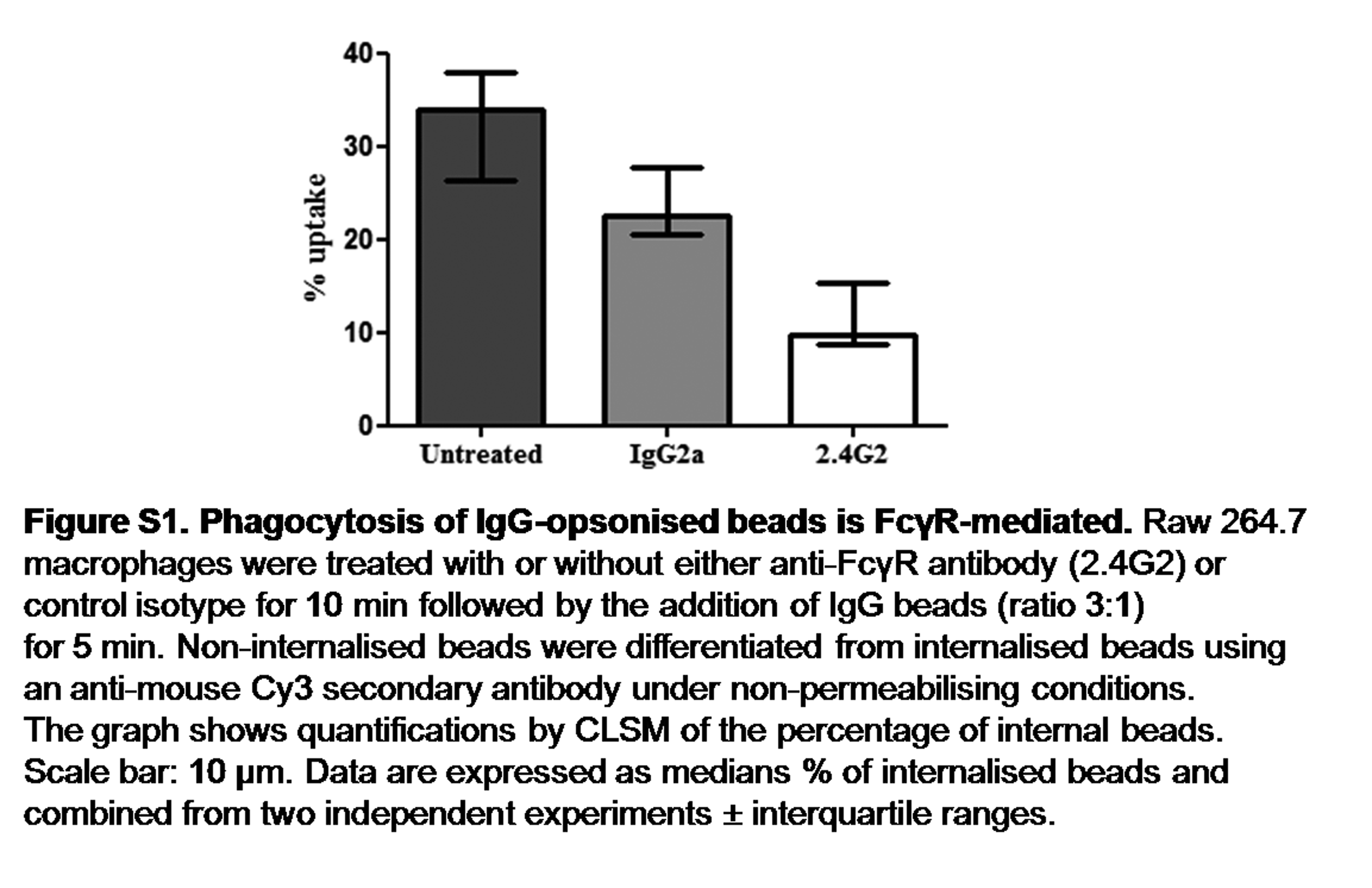

Supplement: S1 Fig — Raw 264.7 macrophages were treated with or without either anti-FcγR antibody (2.4G2) or control isotype for 10 min followed by the addition of IgG beads (ratio 3:1) for 5 min. Non-internalised beads were differentiated from internalised beads using an anti-mouse Cy3 secondary antibody under non-permeabilising conditions. The graph shows quantifications by CLSM of the percentage of internal beads. Scale bar: 10 μm. Data are expressed as medians % of internalised beads and combined from two independent experiments ± interquartile ranges. (TIF) [file pone.0174973.s001.tif]

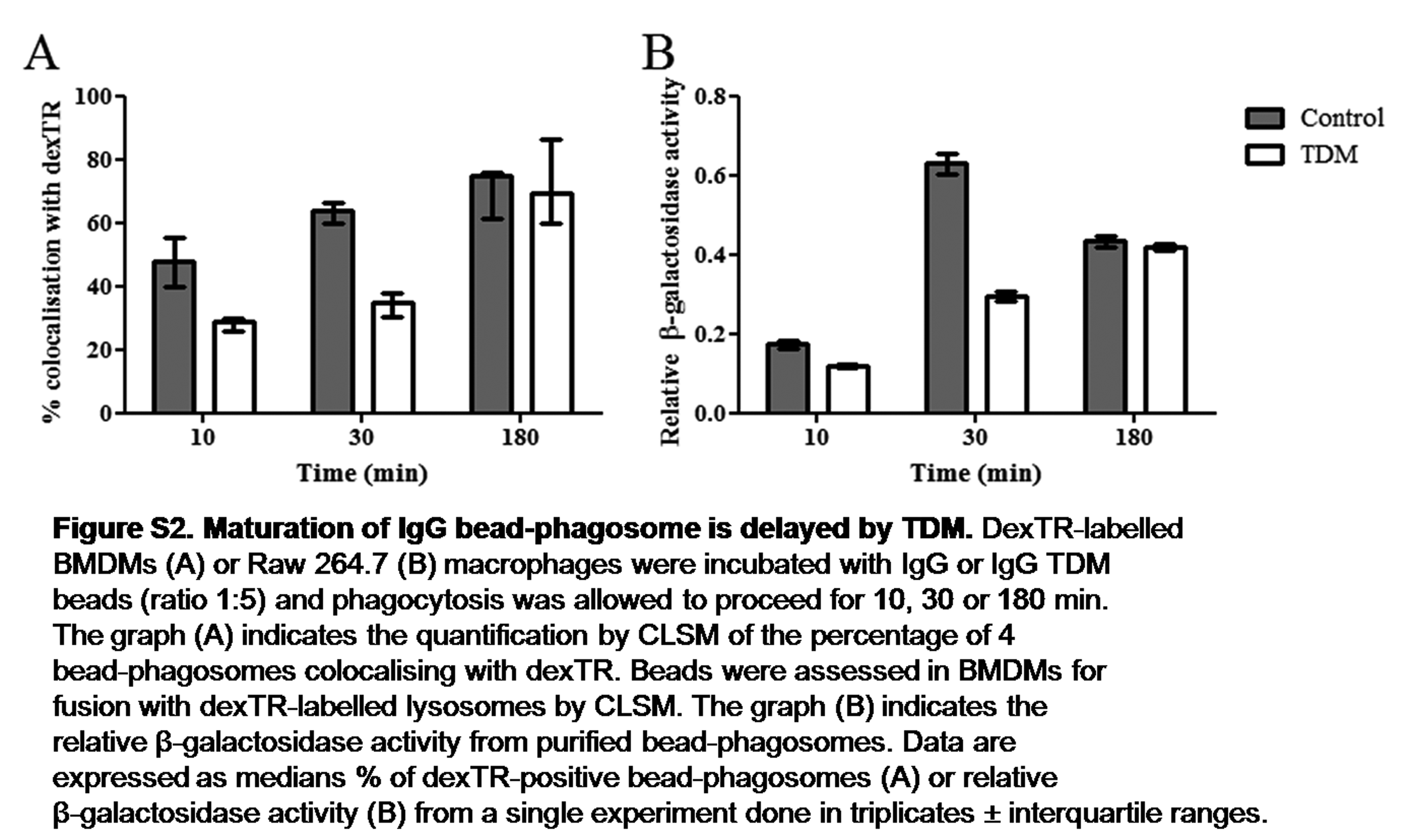

Supplement: S2 Fig — DexTR-labelled BMDMs (A) or Raw 264.7 (B) macrophages were incubated with IgG or IgG TDM beads (ratio 1:5) and phagocytosis was allowed to proceed for 10, 30 or 180 min. The graph (A) indicates the quantification by CLSM of the percentage of bead-phagosomes colocalising with dexTR. Beads were assessed in BMDMs for fusion with dexTR-labelled lysosomes by CLSM. The graph (B) indicates the relative β-galactosidase activity from purified bead-phagosomes. Data are expressed as medians % of dexTR-positive bead-phagosomes (A) or relative β-galactosidase activity (B) from a single experiment each done in triplicates ± interquartile ranges. (TIF) [file pone.0174973.s002.tif]

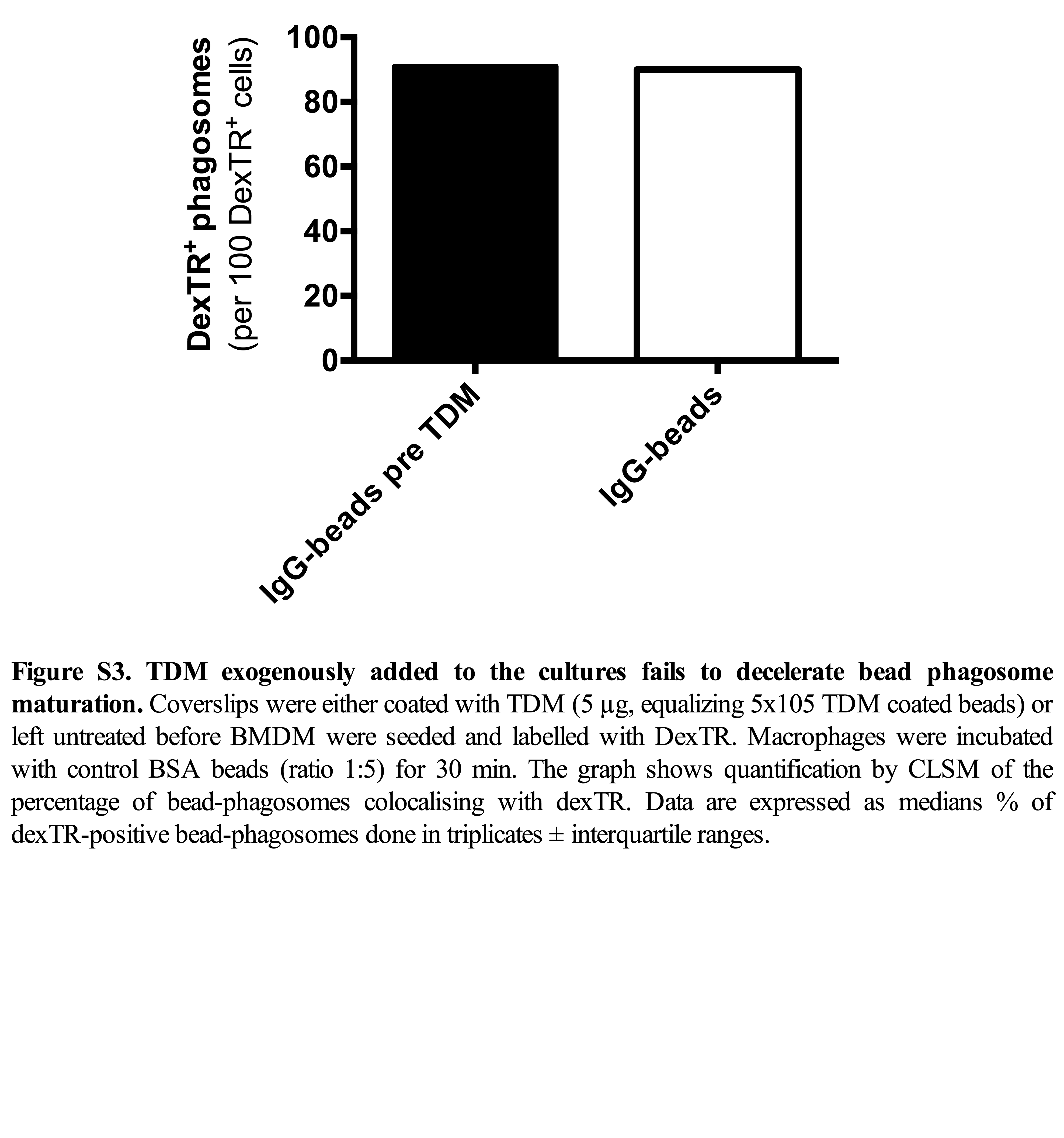

Supplement: S3 Fig — Coverslips were either coated with TDM (5 μg, equalizing 5x10^5^ TDM coated beads) or left untreated before BMDM were seeded and labelled with DexTR. Macrophages were incubated with control BSA beads (ratio 1:5) for 30 min. The graph shows quantification by CLSM of the percentage of bead-phagosomes colocalising with dexTR. Data are expressed as medians % of dexTR-positive bead-phagosomes done in triplicates ± interquartile ranges. (TIFF) [file pone.0174973.s003.tiff]

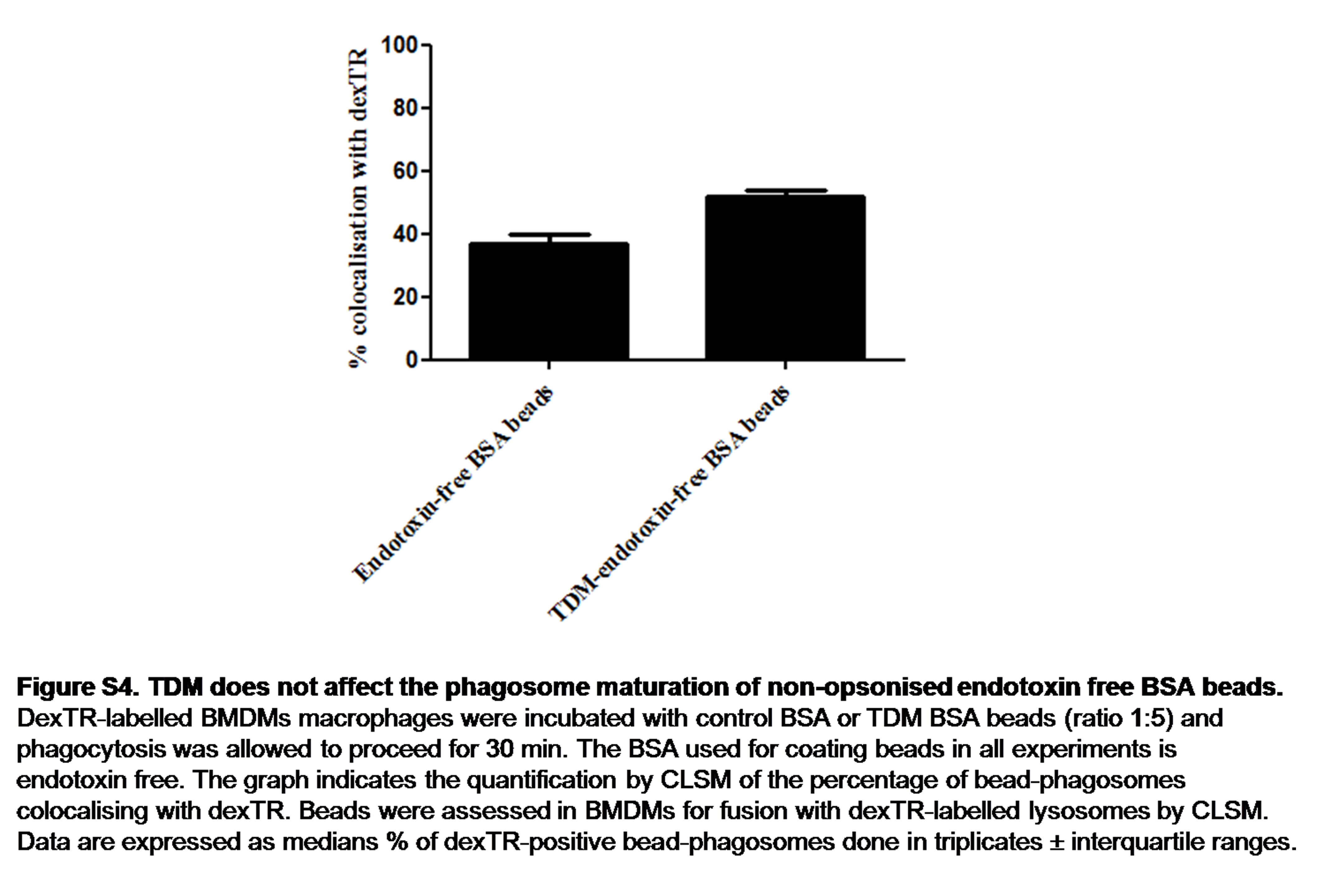

Supplement: S4 Fig — DexTR-labelled BMDMs macrophages were incubated with control BSA or TDM BSA beads (ratio 1:5) and phagocytosis was allowed to proceed for 30 min. The BSA used for coating beads in all experiments is endotoxin free. The graph indicates the quantification by CLSM of the percentage of bead-phagosomes colocalising with dexTR. Beads were assessed in BMDMs for fusion with dexTR-labelled lysosomes by CLSM. Data are expressed as medians % of dexTR-positive bead-phagosomes done in triplicates ± interquartile ranges. (TIF) [file pone.0174973.s004.tif]
