# Supplementary material for: Trehalose dimycolate interferes with FcγR-mediated phagosome maturation through Mincle, SHP-1 and FcγRIIB signalling
Source: PLoS One. 2017 Apr 6;12(4):e0174973. doi: 10.1371/journal.pone.0174973 (PMC5383150; doi:10.1371/journal.pone.0174973)
Supplement: S1 File — Experimental procedures: 1. Assessment of bead phagocytosis; 2. Isolation of bead-phagosomes; 3. SHP-1 expression analysis by Western blot (DOC) [file pone.0174973.s005.doc]

**Supplementary materials**

**Experimental procedures**

**Assessment of bead phagocytosis**

Raw 264.7 macrophages were seeded at 10^5^ cells per well in triplicates on chamber slides (Lab Teck II Chamber Slides, Fisher, UK). To block FcγR uptake, 10mg/ml rat anti-mouse Fcγ III/II receptors (2.4G2, BD Biosciences, UK) or rat isotype control IgG2a (BD Biosciences, UK) were added to Raw 264.7 macrophages for 10 min at 4°C in serum-free medium prior to addition of beads. Macrophages were challenged with beads at a multiplicity of 3 beads/cell in triplicates. To synchronize uptake, beads were attached to macrophages using a magnet and subsequently incubated for 5 min at 37°C, 5% humidified CO_2_. Cells were immediately fixed with 2% PFA for 15 min at RT. To detect non-internalised IgG-opsonised beads, cells were incubated under non-permeabilising conditions for 1 h at RT in blocking buffer containing 5% goat serum (GS) in PBS followed by staining with goat anti-mouse Cy3 (Jackson Immuno Research, USA) diluted 1/200 in blocking buffer for 30 min at RT. Cells were fixed with 2% PFA for 15 min at RT. Chamber slides were mounted with an aqueous-based mounting medium (Fluoromount, Sigma-Aldrich, UK) for CLSM analysis. Percentages of beads inside the cells were determined by manually counting beads unstained for anti-mouse Cy3 (i.e., internalised) in at least 50 macrophages per replicate.

**Isolation of bead-phagosomes**

Raw 264.7 macrophages were seeded at 10^7^ cells/T75 flasks. Beads were added to the monolayer of macrophages at a multiplicity of 5 beads per cell in triplicates for 5 min at 4°C to synchronize uptake and subsequently for 5 min at 37°C, 5% humidified CO_2_. Cells were washed with fresh D10 warmed at 37°C to remove excess beads and phagocytosis was allowed to proceed for the designated amount of time in fresh medium at 37 °C, 5% humidified CO_2_. Cells were harvested by centrifugation at 300g, 5 min at 4°C. Pellets were resuspended in 1 ml Homogenisation Buffer (HB) containing 250mM sucrose; 20mM HEPES pH 7.0, 1% gelatin and protease inhibitors (Complete Mini®, Roche, UK). Bead-phagosomes were isolated from cells by disrupting them using a metal Dounce homogenizer at 4°C. DNA was subsequently broken up by 5 passages through a 23 gauge needle. Bead-phagosomes were washed in HB using the magnet and incubated with 50 U/ml of DNAse (Benzonase®, Merck, UK) for 10 min at 37°C. The bead-phagosomes were washed in HB, layered onto 2 ml 15% Ficoll (PM70) and centrifuged at 500 g for 10 min at 4°C. The pellet of phagosomes were washed 3 times in HB and resuspended in 100 µl PBS. 10 µl of bead-phagosome preparation were analysed for lysosomal β-galactosidase activity.

**SHP-1 expression analysis by Western blot**

In parallel with corresponding phagosome maturation studies, WT or Mincle KO BMDMs were seeded at 2.5×10^6^/well on 6-well flat bottom plates in triplicate and stimulated with 100 ng/ml LPS overnight at 37°C, 5% humidified CO_2_ or left untreated. BMDMs were lysed in ice-cold cell lysis buffer containing 2% Triton X-100, 10 mM Tris-HCl, 150 mM NaCl, 2 mM EDTA, protease inhibitors (Complete Mini®, Roche, UK) for 1 h at 4°C. Lysates from the different replicates were pooled together, centrifuged at 2800 g, 5 min at 4°C and supernatants were collected. As indicated, equivalent protein amounts or bead numbers were loaded on a 10% SDS poly-acrylamide gel. Proteins were transferred to nitrocellulose using the wet technique according to the manufacturer’s instructions (Mini Trans-Blot® Electrophoretic Transfer Cell, Bio-Rad). Membranes were blocked and incubated with primary rabbit anti-mouse SHP-1 (C-19, Santa Cruz Biotechnology, USA) or rat anti-Mincle (4A9, MBL International, USA) antibodies or directly incubated with secondary antibodies for IgG bead preparations. Primary rabbit anti-mouse β-actin (Abcam, Germany) was used as a protein loading control. Membranes were then incubated with respective secondary HRP-conjugated goat anti-mouse or anti-rat (Jackson ImmunoResearch) or goat anti-rabbit IRDye 800-conjugated (Rockland) antibodies. Signals were detected using the Odyssey Infrared Imaging System fluorometer (LI-COR) or the ECL Advance Chemiluminescence kit and ECL Hyperfilm (GE Healthcare, UK) according to the manufacturers’ instructions.
